# Growth inhibition of *Trichophyton rubrum* by laser irradiation: exploring further experimental aspects in an *in vitro* evaluation study

**DOI:** 10.1186/s12866-022-02726-4

**Published:** 2022-12-19

**Authors:** Ruina Zhang, Junying Zhao, Linfeng Li

**Affiliations:** Department of dermatology, Beijing friendship hospital, Capital medical university, 95 Yongan Road, Xicheng District, 100050 Beijing, China

**Keywords:** *Trichophyton rubrum*, onychomycosis, 1064-nm Nd:YAG laser, irradiation area, irradiation energy

## Abstract

**Background:**

According to the results of the clinical trials, laser therapy is effective for the treatment of onychomycosis, but the *in vitro* findings are inconsistent among studies. This study aimed to explore the experimental conditions of laser for the inhibition of *Trichophyton rubrum* growth *in vitro*. A 1064-nm neodymium-doped yttrium aluminum garnet (Nd:YAG) laser was used to irradiate colonies using a small (6-mm diameter) or large (13-mm diameter) area, and using 300, 408, or 600 J/cm^2^. The surface temperature of the colony was measured after irradiation and every 2 min. The growth area was calculated until the 7th or 10th day of incubation daily.

**Results:**

For the small area group, at 300 J/cm^2^, the immediate surface temperature was 25.2 ± 0.2°C, but without effect on growth (*P* = 0.516). At 408 J/cm^2^, the immediate surface temperature was 32.0 ± 0.4°C; growth was inhibited for 7 days (*P* < 0.001). At 600 J/cm^2^, the immediate surface temperature was 38.1 ± 0.4°C; the growth was completely stopped for at least 10 days (*P* < 0.001). For the large area group, the temperature patterns were similar to those of the small area group, but the highest temperature was lower than in the small area groups, and no growth inhibition effect was observed (all *P* > 0.05).

**Conclusions:**

When the irradiation area is small, a 1064-nm Nd:YAG laser at 408 or 600 J/cm^2^ can be effective in suppressing *T. rubrum* growth *in vitro*.

**Supplementary Information:**

The online version contains supplementary material available at 10.1186/s12866-022-02726-4.

## Introduction

The 1064-nm neodymium-doped yttrium aluminum garnet (Nd:YAG) laser is commonly used in medicine for ophthalmologic corrections, thermotherapy for a variety of malignant and benign skin conditions, surgery of the thyroid, liver, uterus, prostate lesions, and in cosmetic dermatology for hair removal [1–4]. This laser has a number of advantages such as long wavelength, high energy density, simple operation, skin penetration of up to 3.1 mm [5], and no mutagenic effect on cellular DNA [6]. It is also the most widely used laser for the treatment of onychomycosis [6, 7].

Onychomycosis is a common fungal infection of the nails (mostly toenails) with an overall prevalence of 2%-13%, but with a markedly higher prevalence in the elderly (50% among individuals > 70 years of age), patients with HIV infection (30%) and patients with diabetes (34%) [8]. Onychomycosis is caused by dermatophyte, non-dermatophyte and yeast, among which *Trichophyton rubrum (T. rubrum)* is the most common pathogeny, accounting for about 90%. A large number of studies confirmed that the 1064-nm Nd:YAG laser is effective against onychomycosis [6, 9–11]. In the study by Kim *et al*. [6], 174 nails were treated using a 1064-nm Nd:YAG laser at 200 mJ pulse energy, 1.5-mm spot size, and 30 Hz; the Onychomycosis severity index(OSI) decreased significantly after 4–6 laser treatments. In the study by Wanitphakdeedecha *et al*. [10], 64 onychomycosis nails (35 patients) were treated with a long-pulsed 1064-nm Nd:YAG laser in four sessions at 1-week intervals, using 4-mm spot size with 35–45 J/cm^2^, pulse duration of 30–35 ms and frequency of 1 Hz; the overall cure rates at 1, 3 and 6 months were 63.5%, 57.7%, and 51.9%, respectively. In the study by Zalacain *et al*. [11], 256 infected toenails were treated with a 1064-nm laser (1 Hz, 35–40 J/cm^2^, 30 W, and sweeping an affected area of 3 mm in diameter); after 3 months, five patients were completely symptom-free with negative culture. In 25 patients, the absence of symptoms was achieved at 6 months with negative cultures.

Some authors believe that the laser treatment of onychomycosis is achieved through the principle of "selective photo-thermal effect", i.e., a combination of light, heat and impact effects can achieve fungal inhibition or killing [12]. Yue *et al*. [13] observed the ultrastructural changes in the hyphae using electron microscopy after irradiating *T. rubrum* with a laser. They found that the double-layer cell wall and organelle structures of the fungus after laser irradiation were blurred or damaged. Lipid droplets, vacuole-like degeneration, myeloid bodies, and protein coagulum could be seen. Nevertheless, the results *in vitro* are conflicting [14–17]. Hence, the aim of the present study was to explore the optimal experimental conditions for the inhibition of *T. rubrum* growth using a 1064-nm Nd:YAG laser.

## Material and methods

### Preparation of the fungal suspension

*T. rubrum* that we used in this study was a clinical strain, taken from the toenail of a patient, who had a good response to laser treatment. This strain was inoculated on Sabouraud dextrose agar (SDA; #PBO19A, Beijing Ginko-Forest Science Co., Ltd., Beijing, China) at 25°C for activation and purification for 8 days.

Sterile saline solution (0.85%, 1 ml, Sigma, USA) was added into the culture dish, gently aspirated with a Pasteur pipette using a single-channel pipettor, transferred to a sterile test tube, and left for 3–5 minutes. The upper liquid (including conidia and hyphae) was removed, shaken for 15 s, and placed in a turbidimetric tube to determine the concentration using a turbidimeter (bioMerieux, France).

Different concentration of fungal suspension, volume and incubation time were needed to be considered before the test, so we performed multiple test conditions before given laser irradiation. If the concentration was too low, and the inoculum volume was too large, it is difficult to form a single colony. If the concentration was too high, it could form a single colony, but the area was too large, and irradiation was difficult, etc (Supplementary Table S1). Finally, one microliter of fungal suspension with 1.0 MacFarland (Mcf) was used to form single colonies. The fungal suspensions were separately inoculated on SDA plates on two sites; one side was used as a negative control, and the other side was used for laser irradiation. The plates were incubated at 25°C until colonies were visible (7–10 days).

### Laser irradiation

**Fig. 1:**
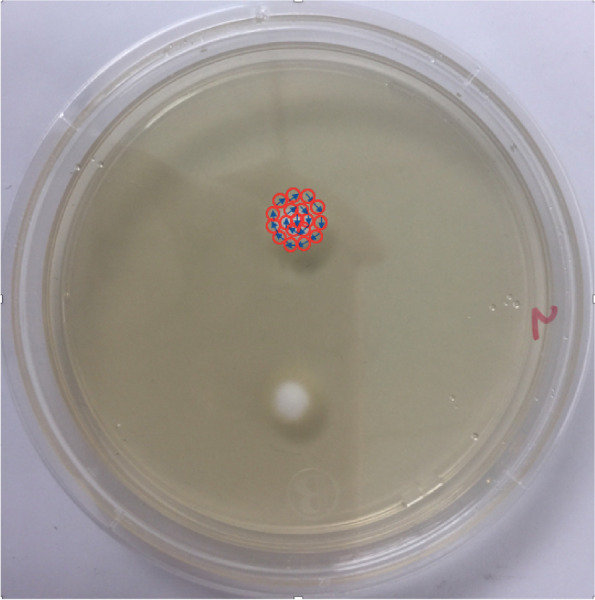
Laser scanning in the spiral pattern over the colony and repeated till 200 spots

The plates were divided into two groups according to the area to be irradiated: the small area (6-mm diameter) group and the large area (13-mm diameter) group. The laser instrument was a long-pulse 1064-nm Nd:YAG laser (Beijing Shiji Guangtong Biotechnology Co., Ltd.). The light spot diameter was 3 mm. The frequency was 1 Hz. The pulse width was 30 ms. Both groups were divided into three subgroups according to laser energy: 300, 408, and 600 J/cm^2^. Based on a previous study [18], 300 J/cm^2^ is similar to the energy used clinically, 408 J/cm^2^ is a little higher than the clinical energy, and 600 J/cm^2^ is significantly higher than the clinical energy. Laser irradiation was administrated in a spiral pattern with a total of 200 spots (as shown in Fig. 1).

The immediate surface temperature of the colony was measured after irradiation and every two min until the initial temperature was restored. The temperature was measured with an infrared thermometer (FLUKE MT4 MAX+), and three points were used each time to take the average value. Three parallel controls were set for each group.

The colonies were daily photographed from the first day of laser irradiation, using an iPhone Plus6 at 8 million pixels, mounted on a tripod that was not moved. Therefore, all pictures were taken at the same location and using the same light source. The distance between the camera and the culture medium was about 10cm. The diameter of each colony was measured with a ruler at the same time every day, at the bottom of the plate; the diameter was the edge of the colony. Measurement with a ruler can be accurate to 0.5 mm. Then, the formula S = π(d/2)² was applied to calculate the colony area. The small area group was irradiated at day 7, while the large area group was irradiated at day 10.

### Statistical analysis

The statistical analyses were performed using Graphpad Prism 6.0 (GraphPad Software Inc., San Diego, CA, USA). The colony areas before and after laser irradiation and the temperatures were expressed as means ± standard deviations (SD). The time effect and inter-group effect were compared by repeated-measures ANOVA, followed by the Bonferroni post hoc test. Normality and homogeneity of variances were tested using the Kolmogorov-Smirnov test and the Levene test, respectively. Two-sided *P*-values < 0.05 were considered statistically significant.

## Results

### One-time irradiation with 200 spots in the small area group

**Fig. 2:**
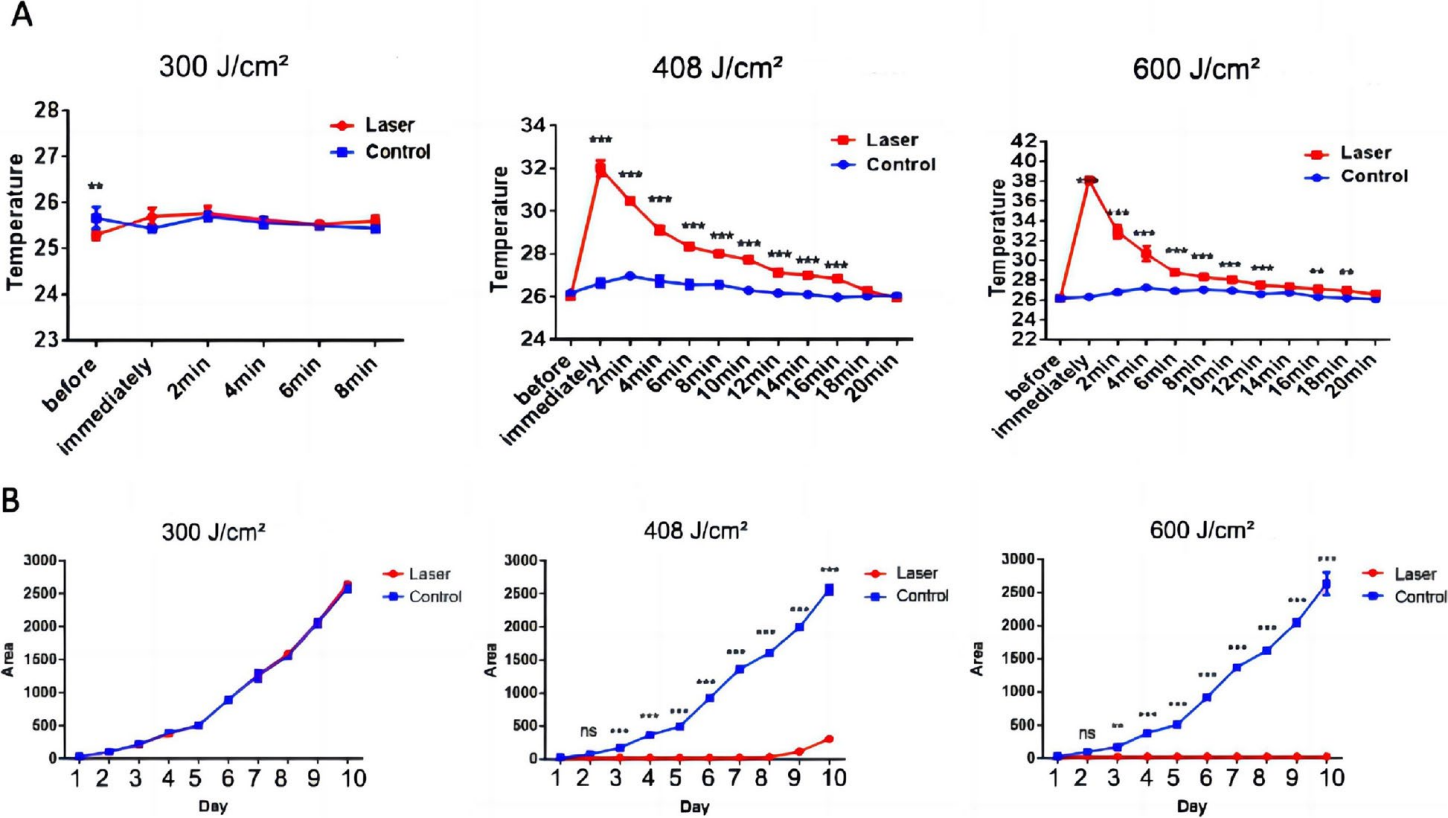
Effect of a long-pulse 1064-nm Nd:YAG laser small-area irradiation (300, 408 and 600 J/cm^2^; 200 spots) on the surface temperature and growth of *T. rubrum in vitro*. **A** The surface temperature (°C) of the colony before laser irradiation immediately after laser irradiation, and then every two minutes. **B** Growth area (mm^2^) of the colonies was measured. *P<0.05，**P<0.01，***P<0.001

**Fig. 3:**
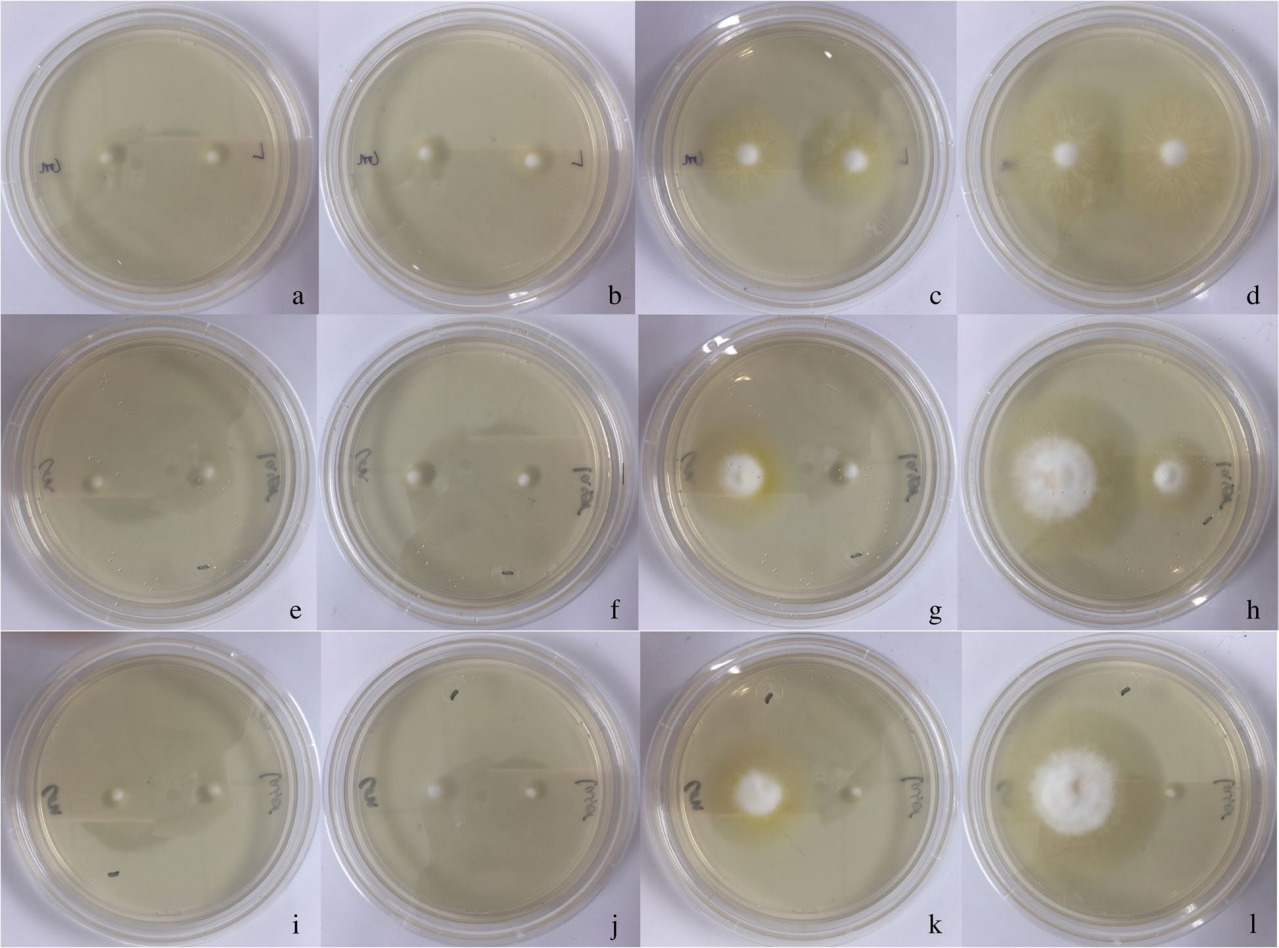
Effect of a long-pulse 1064-nm Nd:YAG laser small-area irradiation (300, 408 and 600 J/cm^2^; 200 spots) on the growth of *T. rubrum in vitro*.At 300 J/cm^2^, colony area was not different from that control until the 9th day. At 408 J/cm^2^, *T. rubrum* was inhibited，growth started again on the 8th day and was gradually restored. At 600 J/cm^2^, the colony growth was completely stopped. *T. rubrum* colonies (left side: control; right side: laser). a-d: 300 J/cm^2^, colonies on the first, second, sixth, and ninth days, respectively; e-h: 408 J/cm^2^, colonies on the first, second, eighth, and tenth days, respectively; i-l: 600 J/cm^2^, colonies on the first, second, eighth, and tenth days, respectively

Figures 2 and 3 show the temperature curve and colony growth after irradiation in the small area group. The baseline temperature (*T.rubrum* growth temperature) was 25 ± 0.5°C. At 300 J/cm^2^, the immediate surface temperature of the colony after a single irradiation was 25.7 ± 0.2°C, and the baseline temperature was restored after 8 min (Fig. 2A). At the end of incubation, the colony area was not different from that control (*P* = 0.516) (Fig. 2B). At 408 J/cm^2^, the surface temperature of the colony was 32.0 ± 0.4°C, which was reduced to 30.5 ± 0.1°C in 2 min, and the initial temperature was restored in 20 min. *T. rubrum* was inhibited (*P* < 0.001): growth started again on the 8th day, and growth was gradually restored. At 600 J/cm^2^, the immediate temperature was 38.1 ± 0.4°C after a single irradiation, and the baseline temperature was restored in 20 min. The colony area was observed every day after laser irradiation until the 14th day, and growth was completely stopped (*P* < 0.001).

### One-time irradiation with 200 spots in the large area group

**Fig. 4:**
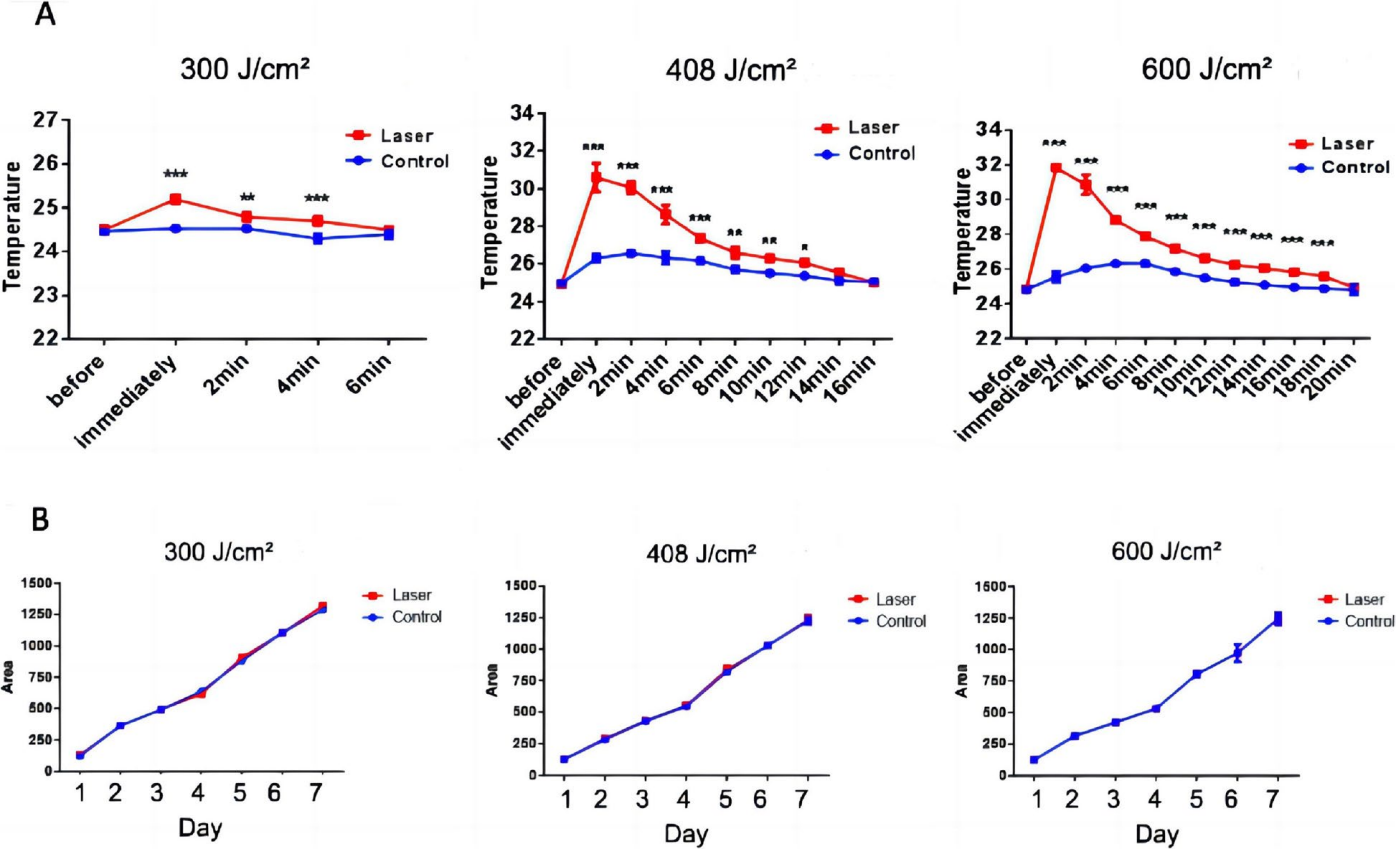
Effect of a long-pulse 1064-nm Nd:YAG laser large-area irradiation (300, 408 and 600 J/cm^2^; 200 spots) on the surface temperature and growth of *T. rubrum in vitro*. **A** The surface temperature (°C) of the colony before laser irradiation immediately after laser irradiation, and then every two minutes. **B** Growth area (mm^2^) of the colonies was measured. *P<0.05，**P<0.01，***P<0.001

**Fig. 5:**
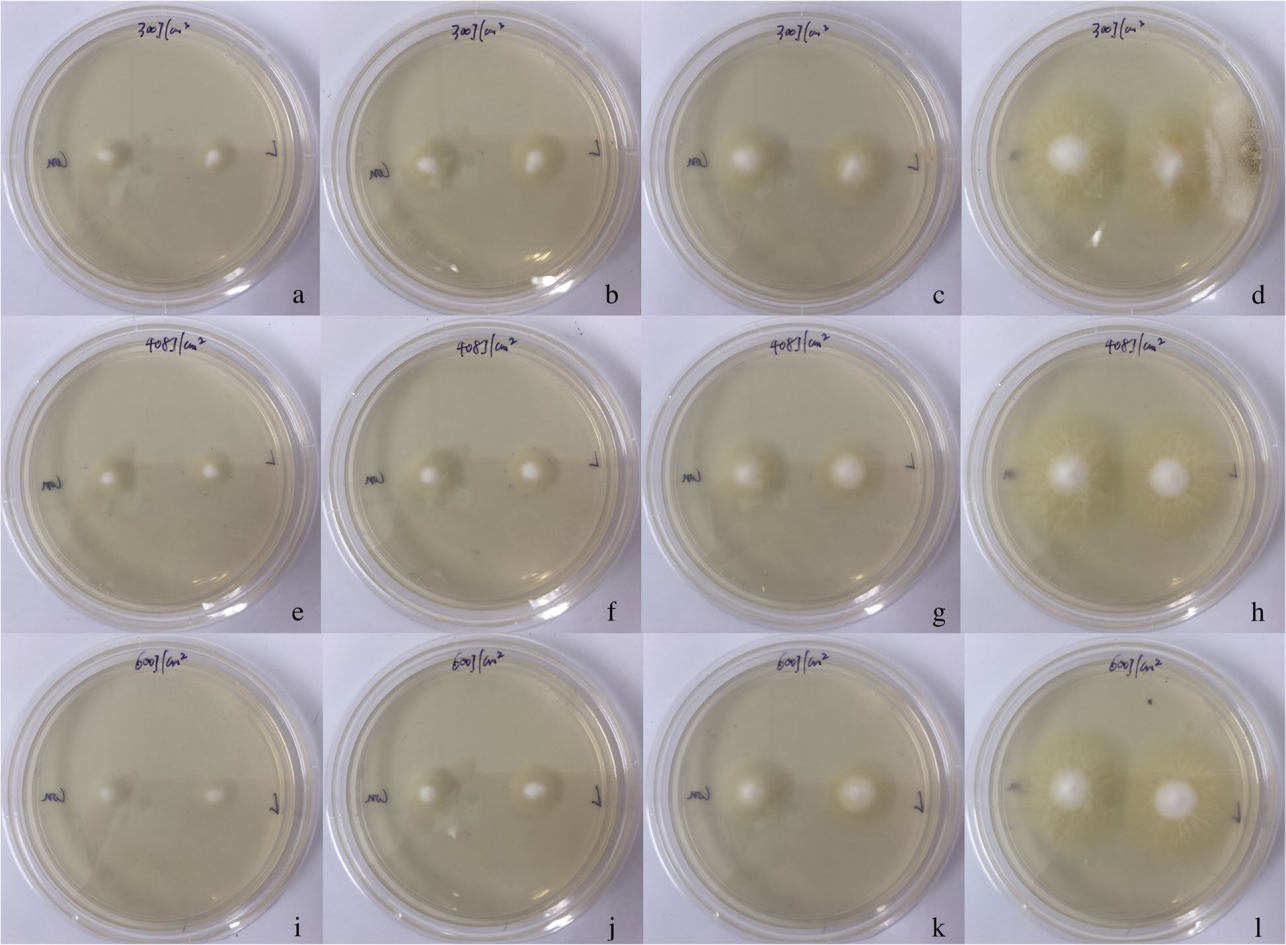
Effect of a long-pulse 1064-nm Nd:YAG laser large-area irradiation (300, 408 and 600 J/cm^2^; 200 spots) on the growth of *T. rubrum in vitro*. There was no significant inhibitory effect on the large-area colony when the energy density was 300, 408 and 600 J/cm^2^

The initial temperature was 25 ± 0.5°C, as in the small area group. Figures 4 and 5 present the temperature curve and colony growth after irradiation in the large area group. The temperature patterns were similar to those of the small area group, with immediate surface temperatures of 25.2 ± 0.1°C, 30.6 ± 0.8°C and 31.8 ± 0.2°C, in the 300, 408 and 600 J/cm^2^ subgroups, respectively (Fig. 4A). The initial temperatures were restored in 6, 16, and 20 min, respectively. There was no effect on growth (all *P* > 0.05) (Fig. 4B).

*T. rubrum* colonies (left side: control; right side: laser). a-d: 300 J/cm^2^, colonies on the first, second, fourth and seventh days, respectively; e-h: 408 J/cm^2^, colonies on the first, second, fourth and seventh days, respectively; i-l: 600 J/cm^2^, colonies on the first, second, fourth and seventh days, respectively.

## Discussion

According to the results of the clinical trials [6, 9–11], laser therapy is effective for the treatment of onychomycosis, but the *in vitro* findings are inconsistent among studies [14–17]. Therefore, this study aimed to explore the experimental conditions of the 1064-nm Nd:YAG laser for the inhibition of *T. rubrum* growth *in vitro*. Our results suggest that when the irradiation area was less than 6 mm, the 1064-nm Nd:YAG laser at 408 or 600 J/cm^2^ can effectively suppress *T. rubrum* growth *in vitro* totally or partially.

*T. rubrum* has a suitable growth temperature of 25–28°C. This study found that at 408 J/cm^2^ with 200 spots over a small area, the growth of *T. rubrum* was inhibited for a week when the temperature of the colony after laser irradiation exceeded the optimal growth temperature of *T. rubrum*. These results may be significant in guiding clinical treatment. Indeed, for the treatment of onychomycosis, when the surface temperature of the infected nails approaches the temperature observed in the present study, or even reaching 40–50°C, as in some studies [19, 20], the growth of the nail fungi should be inhibited for a week. Therefore, the treatment interval should be shortened. Currently, most clinical protocols apply a weekly dose [10, 21, 22]. So according to the study, we speculate whether it is possible to change the clinical protocol and shorten the interval. For example, the laser treatment should be given twice a week and to increase the energy density as much as possible. Using the proper laser, this can be achieved in terms of operability and feasibility, and the only problem may be the higher number of visits to the hospital. Of course, further clinical trials are needed to confirm our assumption. At 300 J/cm^2^ on a small area, the immediate temperature was still within the range of the optimal temperature for fungal growth, so it had not an inhibitory effect. At 600 J/cm^2^, the surface temperature of the colony exceeds the growth temperature of the fungus. While increasing the irradiation area, irrespective of energy, no growth inhibition effect was observed. As for why laser irradiation had no inhibitory effect on the large-area colony, we thought that a single session of laser irradiation cannot make the temperature high enough to inhibit the fungus. Even if when the energy density was 600J/cm^2^, the immediate temperature of the colony only reached 31°C and it dropped to the suitable growth temperature after 4 minutes, so there was no inhibitory effect.

Vural *et al.* [14] used a 1064-nm Q-switched Nd:YAG laser at 4 and 8 J/cm^2^ and a 532-nm Q-switched Nd:YAG laser at 8 J/cm^2^ to irradiate *T. rubrum*, and then observed the growth area of the colonies on the first, third, and the sixth day after irradiation. They found that the growth of *T. rubrum* was significantly inhibited. Ghavam *et al.* [15] found in an *in vitro* study that the laser with a lower energy density, like the red-blue light laser, near-infrared semiconductor laser and laser-diode pumped green laser, did not inhibit the growth of fungi, while the higher power lasers, like the 532-nm Q-switched Nd:YAG laser at 8 J/cm^2^, the Q-switched Nd:AG 1064-nm laser at 4–8 J/cm^2^ and the flashlamp-pumped dye laser at 8–14 J/cm^2^, could inhibit or kill *T. rubrum.* On the other hand, negative results were obtained by Hees *et al.* [16], who irradiated clinical cultures of *T. rubrum* with several types of lasers at different energy (1064-nm Q-switched Nd:YAG laser at 4 and 8 J/cm^2^, 532-nm Q-switched Nd:YAG laser at 8 J/cm^2^ and 1064-nm long-pulsed Nd:YAG laser at 45 and 100 J/cm^2^). Kim *et al.* [17] irradiated five clinical strains of *T. rubrum* with a long-pulse 1064-nm Nd:YAG laser for 0.3 ms at energy of 5 J/cm^2^ on 6 mm, every 3–5 days. On the 29th day, there was no significant difference in the color and area of the colonies, and they concluded that the laser had no inhibitory effect on *T. rubrum.* The lack of agreement on the inhibitory effect of laser on fungi may be because the energy density is not large enough, or the number of pulses is not enough. Therefore, the total energy is not sufficient, so there is no significant impact on the fungus. When using high energy density, it is possible that the number of pulses is sufficient. In the present study, we used a high energy density Nd:YAG laser and a killing effect was achieved at 408 and 600 J/cm^2^, but not at 300 J/cm^2^. Similar clinical effect was observed using higher energy densities [6, 11–13].

The inhibitory effects of laser irradiation on fungi are thought to be related to nonspecific thermal damage and the sensitivity of fungus to produced pigments to light sources. *T. rubrum* contains xanthomegnin, a dominant diffusing red pigment that absorbs laser irradiations at wavelengths of 532 and 598 nm [15]. Therefore, laser irradiation at 532 or 598 nm has inhibitory effects on *T. rubrum* [15]. Although the wavelength of the 1064-nm Nd:YAG laser exceeds the absorption spectrum of xanthomycin, similar inhibitory effects were observed on colonies treated at this wavelength. This may be due to absorption by another chromophore at 1064 nm, e.g., melanin, which is found in the cell wall of conidia [14, 23, 24]. Although *T. rubrum* produces melanin, this study found no obvious pigment production at the early stage, especially when the colonies were 6- or even 13-mm in diameter. With the growth of the colonies, the pigment gradually appeared. These findings indicated that the pigment may not play a critical role in response to laser irradiation, at least *in vitro*, and that the thermal coagulation effect is more important. It can be inferred from this study that the inhibitory effects of laser irradiation on fungi are possibly more associated with energy and exposure time: the higher the energy or the number of light spots administered, the higher the temperature of the fungi. When the temperature exceeds the fungus' tolerance threshold, its growth is inhibited, or death occurs. Therefore, when no pigment is produced, laser irradiation can also exert antifungal effects on *T. rubrum.* The possible mechanism is that high power lasers generate heat and increase the kinetic energy of the target cells, inducing death through evaporation, coagulation, or necrosis [7, 25].

The present study showed that the 1064-nm Nd:YAG laser had an inhibitory effect on *T. rubrum.* The present study and previous negative studies [16, 17] suggest some points for the optimization of the irradiation conditions. First, the *in vitro* environment does not accurately represent the *in vivo* conditions. The culture medium is extremely rich in nutrients, and the numbers of fungi are usually large, which are different from the *in vivo* environment. Although the laser inhibits or kills some fungi at the moment of treatment, the effect doses do not linger, and fungus growth can be restored if there are some fungi left. This is different from the effects of drugs. Second, the colony area at the beginning of laser irradiation may be too large, and a single session of laser irradiation cannot make the surface temperature high enough to inhibit the fungus. Third, the number of fungi on the plate is much larger than that found in infected nails. It was not possible to perform laser irradiation *in vitro* that completely mimics the clinical treatment parameters. Fourth, the infected nails generally have a color, and the therapeutic target of the laser is mostly pigments. The fungus cultured in the medium had not produced significant pigments at the early stage, which could affect laser efficacy. Fifth, the technique or skill of laser irradiation is also a factor. The fungi grow in a radial manner. Therefore, the area to be irradiated needs to be slightly larger than the actual area of the colony. If only the central part is irradiated, the peripheral hyphae are not inhibited.

Taken together, the 1064-nm Nd:YAG laser has an inhibitory effect on *T. rubrum*, but the experimental conditions need to be explored, and they cannot completely be in accordance with the clinically recommended parameters. The factors to be considered include the area of the colony at the beginning of laser irradiation vs. the area that needs to be irradiated (generally, it should be slightly larger than the area of the colony), the laser energy, the number of light spots, the numbers of laser irradiation sessions, and the duration of each irradiation. It was only possible to inhibit the growth of fungi when the cumulative temperature reached a certain amount per unit time and unit area. This is supported by Liu *et al.* [26], who showed that the 1064-nm Nd:YAG laser is effective against *T. rubrum* and that energy density and treatment times are the main factors involved in the effectiveness. Unfortunately, the exact energy output during laser irradiation could not be measured in the present study. Additional studies are still necessary to determine the best conditions for the laser treatment of *T. rubrum*.

## Conclusion

In conclusion, the present study suggests that when the irradiation area is less than 6 mm, the 1064-nm Nd:YAG laser at 408 or 600 J/cm^2^ can be effective in suppressing *T. rubrum* growth *in vitro*.

## Supplementary Information

**Additional file 1: Supplementary Table S1.** Preparatory experiments: colony status under different conditions.

## Data Availability

All data during this study are included in the published article. Meanwhile, the datasets analyzed during the study are available from the corresponding author upon reasonable request.
