# Supplementary material for: Growth inhibition of Trichophyton rubrum by laser irradiation: exploring further experimental aspects in an in vitro evaluation study
Source: BMC Microbiol. 2022 Dec 19;22:307. doi: 10.1186/s12866-022-02726-4 (PMC9762111; doi:10.1186/s12866-022-02726-4)
Supplement: Supplementary file 1 — Additional file 1: Supplementary Table S1. Preparatory experiments: colony status under different conditions. [file 12866_2022_2726_MOESM1_ESM.docx]

**Supplementary Table S1.**

Preparatory experiments: colony status under different conditions

| Concentration | Volume | Days | Results | |
| --- | --- | --- | --- | --- |
| 0.5 Mcf | 10 µl | 7 | The concentration was too low, and the inoculum volume was too large; difficult to form a single colony. |  |
| 1.0 Mcf | 10 µl | 7 | It could form a single colony, but the area was too large, and irradiation was difficult. | |
| 1.0 Mcf | 5 µl | 7 | It could form a single colony, but the area was too large, and irradiation was difficult. | |
| 1.0 Mcf | 1 µl | 7 | It could form a single colony, colony diameter of 6 mm. | |
| 1.0 Mcf | 1 µl | 10 | It could form a single colony, colony diameter of 13 mm. | |
| 0.5 Mcf | 1 µl | 12 | It could form a single colony, but the culture time was too long. | |
